# Glycan-independent binding and internalization of human IgM to FCMR, its cognate cellular receptor

**DOI:** 10.1038/srep42989

**Published:** 2017-02-23

**Authors:** Katy A. Lloyd, Jiabin Wang, Britta C. Urban, Daniel M. Czajkowsky, Richard J. Pleass

**Affiliations:** 1 Liverpool School of Tropical Medicine, Pembroke Place, Liverpool, L3 5QA, UK; 2 Key Laboratory of Systems Biomedicine, Shanghai Jiao Tong University, Shanghai 200240, China; 3 Bio-ID Center, School of Biomedical Engineering, Shanghai Jiao Tong University, Shanghai 200240, China

## Abstract

IgM is the first antibody to be produced in immune responses and plays an important role in the neutralization of bacteria and viruses. Human IgM is heavily glycosylated, featuring five N-linked glycan sites on the μ chain and one on the J-chain. Glycosylation of IgG is known to modulate the effector functions of Fcγ receptors. In contrast, little is known about the effect of glycosylation on IgM binding to the human Fcμ receptor (hFCMR). In this study, we identify the Cμ4 domain of IgM as the target of hFCMR, and show that binding and internalization of IgM by hFCMR is glycan-independent. We generated a homology-based structure for hFCMR and used molecular dynamic simulations to show how this interaction with IgM may occur. Finally, we reveal an inhibitory function for IgM in the proliferation of T cells.

The human Fcμ receptor (hFCMR), also known as Toso or FAIM3, is a high affinity receptor for the Fc portion of human immunoglobulin M (hIgM), which is expressed on B cells, T cells (CD4^+^ and CD8^+^), and a subset of NK cells (CD3^−^CD56^+^)^1^,^2^,^3^. Although the functions of hFCMR are still being resolved, the receptor has been implicated in the homeostasis of IgM in mice^4^,^5^. Mice deficient in FCMR have significantly elevated serum levels of IgM^4^,^5^, and cross-linking of hFCMR on chronic lymphocytic leukaemia (CLL) cells by hIgM results in the rapid internalization of hIgM^6^. Following internalization by hFCMR, hIgM is shuttled through the endocytic pathway to lysosomes and degraded^6^. Although yet to be proven, hFCMR-mediated internalization and degradation of IgM-opsonized antigens may be important for cross-presentation by B cells^1^,^6^. Alternatively, since natural polyclonal IgM is an important first line defense against bacteria and viruses^7^, hFCMR could function to transport hIgM-opsonized immune-complexes to lysosomes where, depending on antigen, TLR activation may ensue. Intriguingly, protein expression and mRNA of hFCMR was reduced in CLL cells following exposure to TLR7 and TLR9 agonists (imiquimod and CpG-ODN), suggesting a link between TLR activation and hFCMR expression^6^.

IgM molecules are heavily glycosylated oligomers containing five N-linked glycosylation sites on each heavy chain and one site on the J-chain^8^. In total, these N-linked glycans constitute approximately 10% of the molecular weight of hIgM^9^. Glycosylation is important for hIgM secretion and its presentation on B cell surfaces^8^,^10^,^11^, yet it is unclear whether hIgM glycosylation is required for binding to hFCMR, and what the functional consequences of this binding may be. Glycosylation of the Fc region of immunoglobulins plays a pivotal role in facilitating the binding to certain high affinity FcRs^12^. Abrogation of IgG glycosylation by mutating the conserved N-linked glycosylation site of IgG (e.g. N297 to N297A by alanine mutagenesis), or by completely removing glycans with the peptide N-glycosidase (PNGase) F, are well established strategies to abrogate binding to FcγRs^13^. Similarily, PNGase F-treatment and mutagenesis of the N-linked site Asn394 in IgE, which is homologous to Asn297 in IgG, results in reduced binding to the high affinity Fcε receptor (FcεRI)^14^,^15^.

In this study, we investigate the effect of hIgM glycosylation on the binding to hFCMR and its subsequent internalization within the cell. Our findings show, surprisingly, that glycosylation of hIgM is not critical for its interaction with hFCMR, which we show is dominated by the Cμ4 domain of hIgM.

## Results

### The Cμ4 domain of hIgM forms the binding site for human FCMR

**Figure 1:**
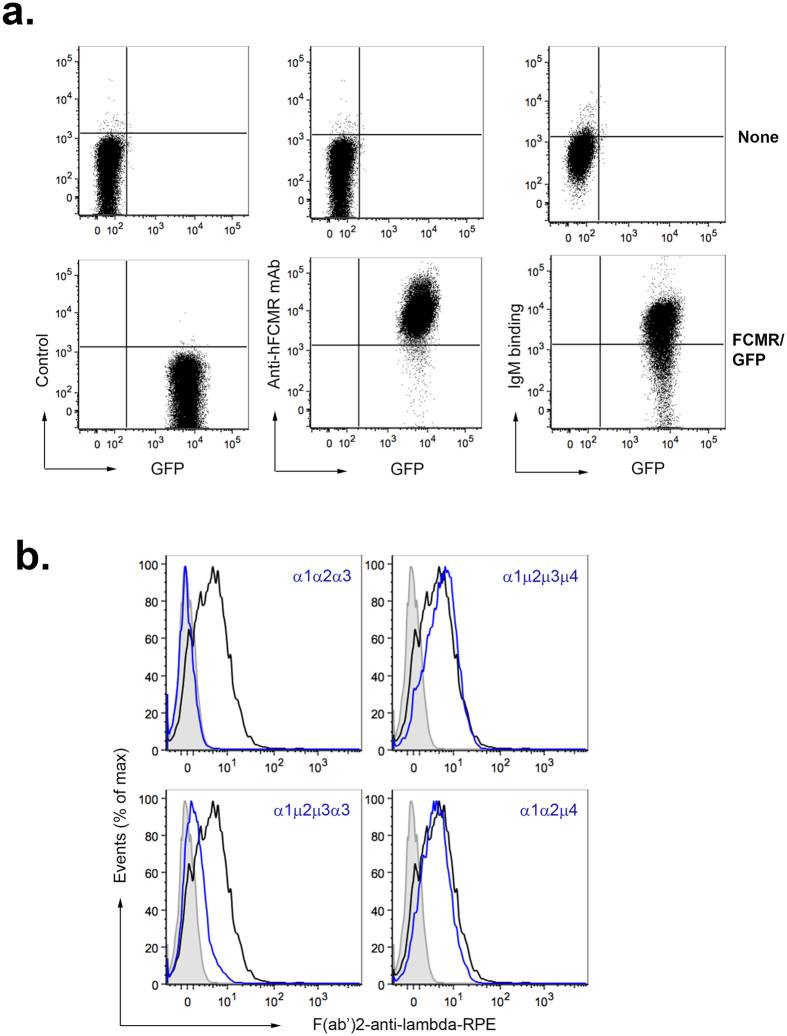
The Cμ4 domain of IgM binds to human FCMR. (**a**) Mouse BW5147 T cell lines alone (none) or transfected with the retroviral construct containing both human FCMR and GFP cDNAs (FCMR/GFP) were incubated with media alone (isotype control; left panels) or media supplemented with mouse anti-hFCMR mAb (1 μg: middle panels), washed and then labeled with anti-mouse IgG-APC to detect hFCMR expression by flow cytometry. The binding of human IgM (15 μg/ml; right panels) to these cell lines was confirmed. (**b**) GFP^+^ hFCMR-transfected cells were incubated with media supplemented with domain-swap Abs (blue trace), hIgM (black trace), or media alone (grey trace) for 1 hr at 4 °C, then washed extensively. Binding of Abs was determined by staining with F(ab’)_2_-anti lambda-RPE and subsequent flow cytometry analysis. Data are representative of three repeat experiments. Domain swap antibodies have been previously described in detail^16^,^17^,^18^.

To determine the region of the IgM molecule critical for interaction with hFCMR, we used a panel of domain-swapped Ab described previously^16^,^17^,^18^, in which homologous constant domains are exchanged between human IgA and IgM. The ability of GFP-gated hFCMR-transfected cell lines^1^ (Fig. 1a) to bind the domain-swapped Ab was analyzed by flow cytometry (Fig. 1b). We observed that those Ab that contained only the Cμ4 domain were able to interact with hFCMR. In contrast, no binding was observed with hIgA and only weak binding was seen with the α1μ2μ3α3 domain-swap lacking the Cμ4 domain. This shows that either the Cμ2 and/or Cμ3 domains are involved in binding hFCMR, although their contribution is less important than the Cμ4 domain.

### Human FCMR is an endocytosis receptor for IgM

**Figure 2:**
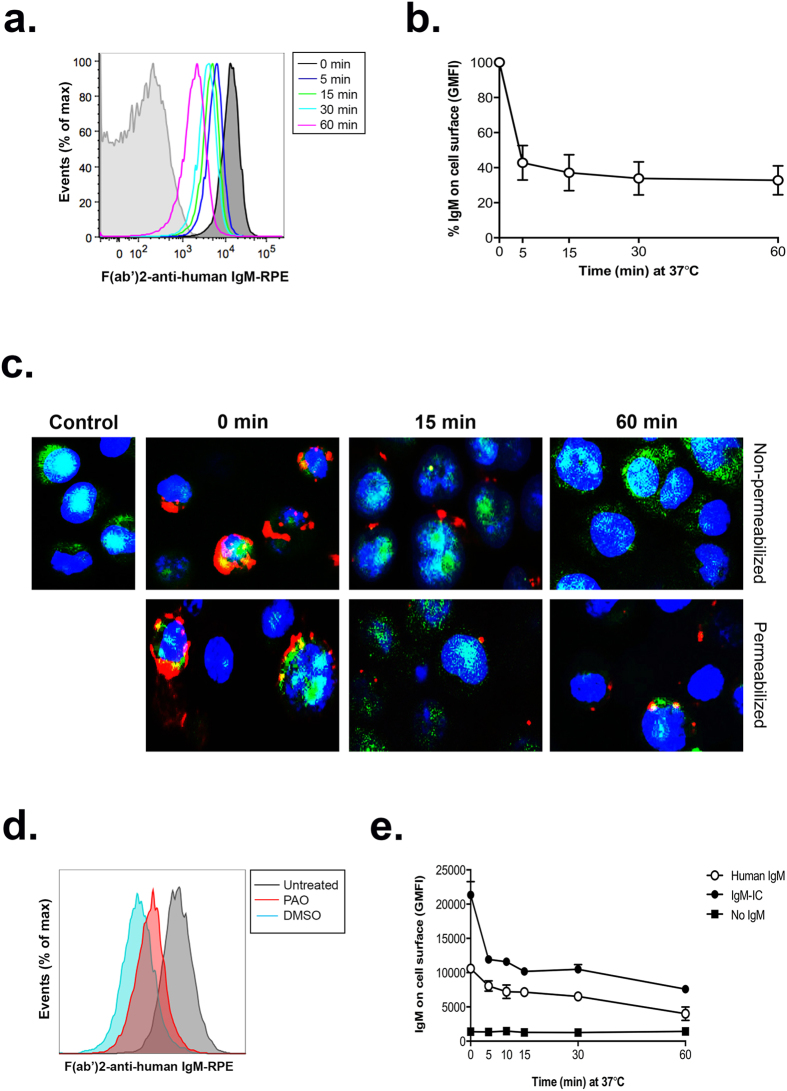
Human FCMR mediates endocytosis of hIgM. (**a**) To investigate hIgM internalization by FCMR, GFP^+^ hFCMR-transfected cells were incubated with media alone (grey trace) or media supplemented with 15 μg/ml purified hIgM for 1 hr at 4 °C. After washing, cells were left at 4 °C (black trace) or incubated at 37 °C for the indicated times. Internalization was halted with the addition of ice-cold media, and cells were washed extensively to remove unbound IgM. Cells were then labeled with F(ab’)_2_-anti-human IgM-RPE and analyzed by flow cytometry. (**b**) IgM cell surface levels (geometric MFI) were normalized to time 0. The mean ± SD from five experiments are shown. (**c**) Following incubation at 37 °C, hFCMR-transfected cells were cytospun, fixed and permeabilized, and incubated with F(ab’)_2_-anti-human IgM-RPE for 1 hr. Cells were washed, and labeled with DAPI prior to visualization. Red labeling depicts IgM levels (anti-hIgM-RPE); blue colour, nuclei (DAPI); and green colour shows hFCMR-transfected cells (GFP). Permeabilization of hFCMR-transfected cells resulted in partial quenching of the GFP signal. (**d**) hFCMR-transfected cells were either left at 4 °C (grey) or incubated for 60 min at 37 °C in the presence or PAO (30 μM, red) or carrier control DMSO (blue). Data shown is one of three representative repeat experiments. (**e**) IgM cell surface levels (GMFI) indicate enhanced binding and internalization of heat-aggregated IgM (IgM-IC) to hFCRM-transfected cell lines, when compared to hIgM (*n* = 3).

We next assessed the ability of hFCMR to internalize hIgM by flow cytometry. hIgM was rapidly internalized into hFCMR-transfected cell lines, with ~50% reduction of hIgM on cell surfaces seen within 5 min of incubation at 37 °C (Fig. 2a,b), and only ~35% hIgM remaining on the cell surface after 1 hr (Fig. 2a,b). The loss of hIgM from the surface of non-permeabilized hFCMR-transfected cells, coupled with the accumulation of hIgM in the cytoplasm of permeabilized hFCMR-transfected cells, following incubations at 37 °C confirmed that hIgM was internalized by hFCMR (Fig. 2c). Furthermore, the well-characterised inhibitor of clathrin-dependent endocytosis phenylarsine oxide (PAO) was used to further confirm and characterise IgM internalization. hFCMR-transfected cells incubated for 60 min at 37 °C in the presence of PAO exhibited reduced IgM internalization when compared to those incubated with DMSO alone (Fig. 2d). These results indicate that hFCMR internalizes IgM at least in part by clathrin-dependent mechanisms. We next tested if hIgM internalization also occurred in the physiological environment provided by human serum, or if other components in human serum affected the uptake of hIgM by hFCMR. There was no significant difference between the internalization of hIgM provided in serum compared to purified hIgM diluted in physiological buffers (P = 0.746, Fig. S1A).

Since immune-complexes (ICs) are known to bind receptors with greater avidity^13^,^19^, we next investigated the effect of IgM-ICs on binding and internalization by hFCMR. Indeed, heat-aggregated hIgM exhibited enhanced binding to hFCMR-transfected cells and was similarly internalized (Fig. 2e).

**Figure 3:**
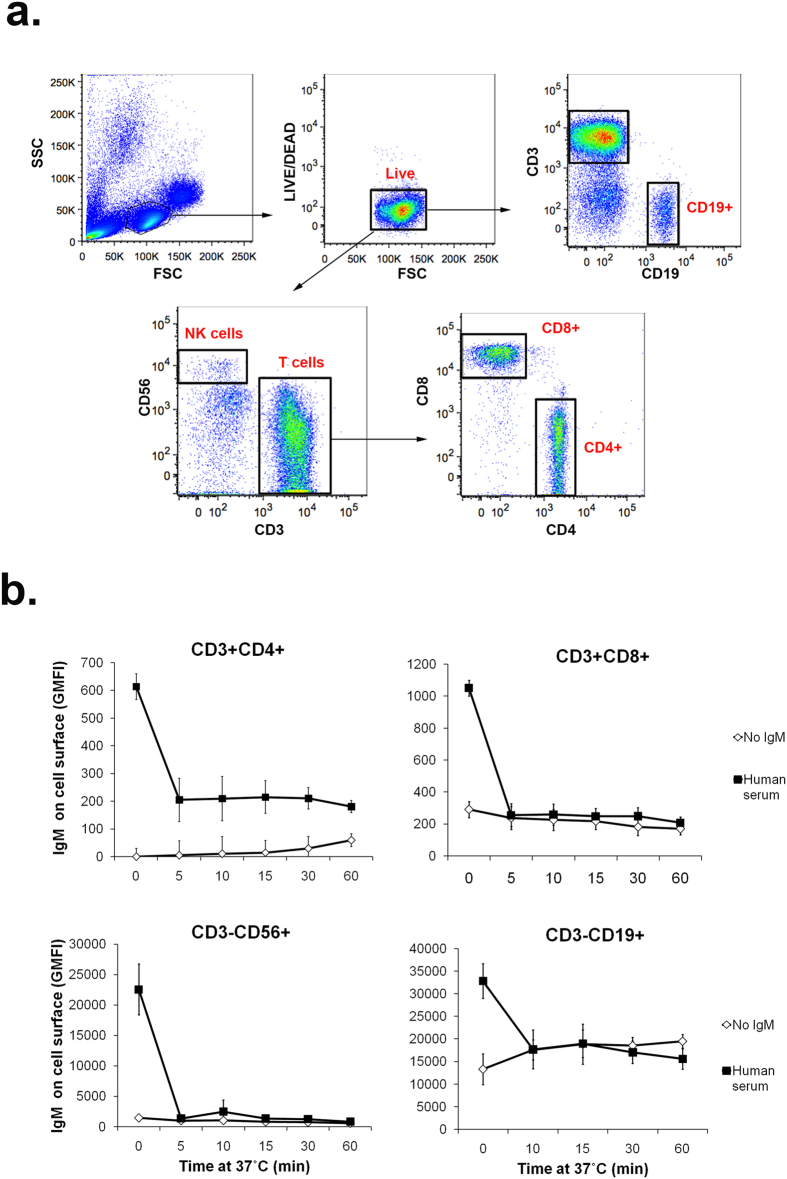
Internalization of IgM on lymphocyte subsets. (**a**) Gating strategy for lymphocyte subsets known to express hFCMR. PBMCs were labeled with 1 μM LIVE/DEAD Aqua Dead Cell stain, anti-human CD3-Brilliant Violet 421, anti-human CD56-Alexa Fluor 700, anti-human CD8-PerCp, and anti-human CD4-FITC for 1 hr at 4 °C. To differentiate B cells, LIVE/DEAD stained PBMCs were incubated with anti-human CD3-Brilliant Violet 421 and anti-human CD19-FITC. Labeled cells were analyzed and gated by flow cytometry. (**b**) Surface levels of IgM (GMFI) on gated lymphocytes incubated with media alone (no IgM) or media supplemented with 10% human serum. The mean ± SD of three independent experiments is shown.

Since cell lines do not reflect potential hFCMR expression on human cells, we next evaluated the ability of lymphocyte subsets known to express hFCMR (Fig. 3a) to bind and internalize hIgM. As shown in Fig. 3b, exogenous hIgM bound to and was internalized by B cells, T cells (CD4^+^ and CD8^+^), and NK cells, in agreement with previous observations^1^. For T cells, CD8^+^ subsets exhibited enhanced internalization of hIgM compared to CD4^+^, with ~65% reduction in hIgM surface levels for CD4^+^ T cells seen within 5 min compared to ~75% reduction for CD8^+^ T cells (Fig. 3b). With NK cells, surface levels of hIgM were reduced by ~85% within 5 min upon incubation (Fig. 3b). Furthermore, there was a noticeable reduction of ~50% of cell surface IgM on B cells upon incubation with human serum compared to those incubated with media alone (Fig. 3b). We observed enhanced rates of hIgM uptake from the surface of all lymphocyte subsets investigated, although the functional consequences of this uptake need to be further studied.

### De-glycosylation does not affect binding or internalization of IgM by hFCMR

The five N-linked glycosylation sites (Asn171, Asn332, Asn395, Asn402, and Asn563) in the μ chain of hIgM are predominantly occupied by complex glycans terminating in sialic acid, galactose, or oligomannose glycans^8^. As glycosylation of human IgG can significantly modulate effector functions via FcγRs, such as antibody-dependent cell cytotoxicity^20^,^21^,^22^, we hypothesized that de-glycosylation of hIgM may also affect interactions with hFCMR.

**Figure 4:**
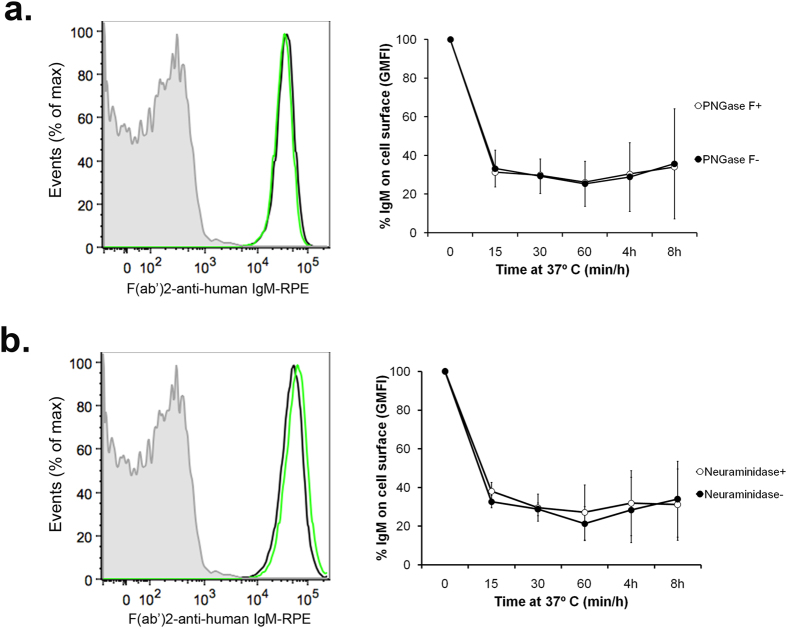
Effect of de-glycosylation on FCMR-mediated IgM internalization. PNGase F (**a**) and neuraminidase (**b**) were used to cleave glycans from IgM molecules. The binding of endoglycosidase-treated (green trace) and non-treated (black trace) hIgM to hFCMR-transfected cells is shown as traces in the left panels. Internalization of endoglycosidase-treated IgM was also investigated, whereby IgM surface levels (GMFI) were normalized to time 0 (right panels). The mean ± SD of three independent repeats is shown.

N-linked glycans were removed from hIgM with PNGase F or neuraminidase (Fig. S2), and the ability of de-glycosylated or de-sialylated hIgM to bind hFCMR was investigated. De-glycosylation and de-sialylation of IgM was confirmed with Coomassie staining and immunoblotting with either SNA lectin or anti-human IgM (Fig. S2B). The de-glycosylation or de-sialylation of hIgM with either PNGase F or neuraminidase respectively had no obvious effect on binding or internalization of hIgM by hFCMR-transfected cell lines, when compared to non-treated hIgM (Fig. 4a and b, respectively). Intriguingly, de-glycosylation of IgM with PNGase F reduced its binding to untransfected cells, whereas neuraminidase-treatment had a lesser effect (Fig. S3). This data suggests that the binding of IgM to untransfected cells, although minimal compared to IgM binding to hFCMR–transfected cells, is most likely also owing to scavenging lectin interactions.

Lymphocytes express numerous receptors known to bind glycans, including C-type lectin receptors including CD62L, CD33 (Siglec-3), or CD22 (Siglec-2)^23^,^24^,^25^,^26^. Therefore, to rule out the involvement of glycan receptors in hIgM internalization by lymphocytes, internalization assays were repeated with de-glycosylated hIgM (Fig. S4). There was no difference in the internalization of de-glycosylated hIgM by T cells (CD4^+^ and CD8^+^) and B cells, when compared to non-treated hIgM (Fig. S4). Together, these results demonstrate that PNGase F or neuraminidase susceptible glycan(s), do not likely contribute to the binding or internalization of hIgM by hFCMR.

### Molecular dynamic stimulations of the interaction between IgM and hFCMR

**Figure 5:**
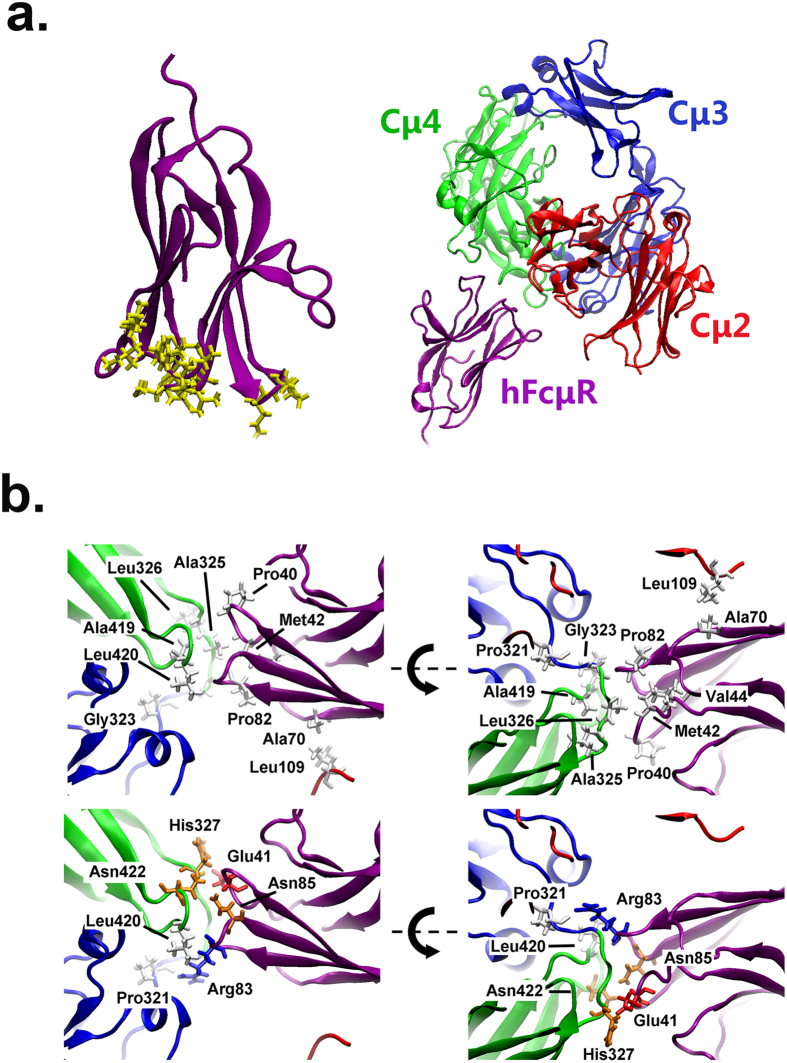
Model of FCMR binding to human IgM. (**a**) Homology model of the monomeric Ig-like domain of hFCMR (left panel). The CDR regions expected to directly contact IgM are shown in stick representation (yellow). Final model of the interaction between the Fc domain of hIgM and hFCMR (right panel). (**b**) Finer details of the IgM/FCMR interface. The residues of one protein that are within 3 Å of the other protein for more than 75% of the simulations are shown in stick representation. The upper panel shows the hydrophobic residues within this interface, and the lower panel shows the polar residues. The hydrophobic, positively charged, negatively charged, and polar residues are colored white, blue, red, and orange respectively.

To provide structural insight into how IgM may interact with hFCMR, we evaluated potential binding mechanisms using all-atom molecular dynamics (MD) simulations. While there is presently a working atomic model of pentameric IgM^27^, there is currently no structural data available for hFCMR. We thus sought structural homologs of hFCMR using Phyre2, a robust web portal for predicting and analyzing protein structures, functions and mutations^28^,^29^. Two regions of hFCMR were identified that exhibit significant homology to known proteins: one segment (from residues Phe252 to Val272) is homologous to the transmembrane domain of the Erbb2 receptor^30^ and the other segment (from residues Val33 to Gly105) is homologous to many Ig-like domains (Fig. 5a). Two other proteins that bind IgM (namely, FCMR and pIgR) also have similar Ig-like regions, and with these proteins, the Ig-like regions have been shown to directly contact IgM^1^,^31^,^32^,^33^,^34^,^35^. More specifically, this earlier work demonstrated that this interaction occurs via three loops (CDR1, CDR2, and CDR3) at one end of this domain. Thus, we investigated possible binding interactions of the analogous end of the Ig-like domain of hFCMR with IgM by MD simulations.

Based on the FACS results (Figs 1b and 4), we evaluated two putative interactions, namely Cμ2/Cμ4/hFCMR and Cμ3/Cμ4/hFCMR (Supplementary Fig. S5 and methods). Following equilibration of these models (entailing ~30 ns for each system), we found that the number of residues in close contact within the interaction interface and the buried surface area of this interface were both significantly greater in the Cμ2/Cμ4/hFCMR model (Fig. S5). In particular, there were 25 residues within close contact (3 Å) in the Cμ2,4/hFCMR interface but only 18 residues in the Cμ2,3/hFCMR interface. In addition, the area buried within the Cμ2,4/hFCMR interface is 1352 Å^2^ but only 855 Å^2^ within the Cμ2,3/hFCMR interface. This Cμ2,4/hFCMR interfacial area is comparable to the interfacial areas of many oligomeric proteins^36^. Hence, this analysis suggests that the interaction of hFCMR with Cμ2/Cμ4 is more energetically favorable than with Cμ3/Cμ4.

With this, we performed additional simulations of a model of Cμ2/Cμ4/hFCMR (see Methods) to obtain a final structure of this interaction (Fig. 5a). Inspection of the Cμ2,4/hFCMR interface reveals several polar and hydrophobic residues that may play a significant role in mediating this interaction (Fig. 5a). In particular, there were extensive hydrogen-bonding between side chains and backbone atoms involving Asn422, Leu420, Pro321, and His327 in IgM and Pro40, Glu41, Arg83, and Asn85 in hFCMR that surround a hydrophobic pocket consisting of Gly323, Ala325, Leu326 and Ala419 in IgM and Met42, Val44, and Pro82 hFCMR (Fig. 5b). In addition, there is also a smaller hydrophobic patch, involving Leu109 in IgM and Ala70 in hFCMR that also appears to play a significant role in this interaction (Fig. 5b). We note that the Cμ4 N-linked glycan at Asn563 is located in the tailpiece region that is far from this putative binding interface (Fig. S6), consistent with the observation that removal of N-linked glycans does not have a perturbative effect on hFCMR binding (Fig. 4).

### hIgM inhibits T cell proliferation

**Figure 6:**
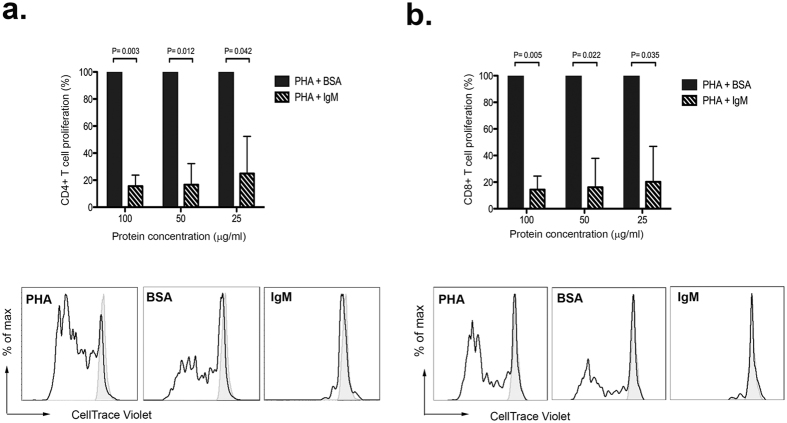
IgM inhibits T cell proliferation. Various concentrations of IgM or BSA were incubated with 5 μg/ml PHA for 30 min at 4 °C, prior to five day incubation with CellTrace Violet-labeled PBMCs at 37 °C. PHA alone was used as a positive control and media only as a negative control (grey trace, lower panels). Percentage of proliferating CD4^+^ and CD8^+^ T cells (panels A and B, respectively) were normalized to samples incubated with PHA and BSA (upper panels). Data are represented as mean ± SD of two independent experiments.

IgM was recently reported to induce potent inhibition of T cell responses^37^. As we showed binding and internalization of IgM by T cells, we wished to determine what effect this has, if any, on T cell proliferation. Peripheral blood mononuclear cells (PBMCs) isolated from healthy donors were stimulated with phytohemagglutinin (PHA) in the presence or absence of hIgM for 5d at 37 °C. Incubations with bovine serum albumin (BSA) were performed in parallel as a negative control. Human IgM strongly inhibited PHA-induced proliferation of CD4^+^ and CD8^+^ T cells in a dose-dependent manner (Fig. 6a and b, respectively). Incubations of PBMCs with hIgM or BSA alone did not induce proliferation of T cells (Fig. S7). No significant inhibition of CD4^+^ and CD8^+^ T cell proliferation was observed with similar concentrations of BSA. Together these results show that hIgM can potently inhibit T cell proliferative responses.

## Discussion

Previous work has shown that the Fc_5_μ fragment of IgM comprised mainly of Cμ3/Cμ4 domains is involved in binding to the hFCMR^1^, although which domain or amino acid residues within the Fc_5_μ that make the critical contacts with hFCMR remained unidentified. Here, we show that the Cμ4 domain of IgM makes the major contribution to binding, with only a minor contribution from the Cμ2 and/or the Cμ3 domains (Fig. 1b). Other receptors for IgM such as hFcα/μR and pIgR also bind the Cμ4 domain^17^,^18^, although they have a very different cellular distribution to the hFCMR.

Since the interaction of IgG with FcγRs is known to be critically dependent on the presence of a N-linked glycan at Asn297 in the Cμ2 domain^38^, and given the presence of an N-linked glycan in the Cμ4 domain of hIgM at position Asn563^8^, we investigated if these glycans contributed to the observed binding with hFCMR (Fig. 4). In contrast to IgG, removal of glycans from hIgM with PNGase F or neuraminidase had no effect on hFCMR binding, although similar treatment of hIgM with PNGase F did significantly reduce interactions with the lectin-dependent receptor DC-SIGN^39^. Similarily, de-glycosylation of human IgA did not affect binding to FcαR (CD89)^40^,^41^, suggesting that glycan-dependent mechanisms are not essential for all antibody binding to FcRs.

The hFCMR has been implicated in IgM homeostasis^1^,^4^,^5^, and one study showed receptor cross-linking with hIgM promoted the rapid internalization of hIgM and hFCMR into CLL B cells, and transfected HeLa and BW5147 cell lines^6^,^42^. Here, we confirm and extend these previous observations by showing that hFCMR also promotes the internalization of IgM complexes (heat-aggregated), and importantly, show that hIgM internalization by hFCMR was glycan independent. In flow cytometry experiments we observed a significant proportion (~40%) of IgM remains on the cell surface while the rest is rapidly internalized. This may occur through heterogeneity of IgM forms (presence or absence of J-chain), or to the expression of different FCMR variants and/or localization within different domains in the plasma membrane. For example, a FCMR splice variant lacking the transmembrane exon has been described that may encode a form of FCMR incapable of internalization^1^. In this regard, we note that for NK cells, the internalization was much more efficient (Fig. 3b), perhaps consistent with a differential ability of FCMR to be internalized in different cells.

These results are supported by recent findings that de-sialylated IgM bound to the surface of T cells^37^. However, this same study found that de-sialylated IgM remained on the cell surface following 24 h incubations at 37 °C, and only sialylated IgM (IgMh1) was internalized and shuttled to lysosomes^37^. Although the receptor responsible for the internalization was not identified in the study, hFCMR involvement was suggested as it is currently the only known receptor for IgM expressed on T cells^37^. We are presently unaware of the reason for these observed differences, although differential expression of hFCMR could offer one explanation for the discrepancy. The receptor is highly expressed on the transfected cells^1^, and previous data has shown dispersed expression on T cell subsets^1^. Furthermore, it is still highly likely that in addition to hFCMR, other glycan receptors for IgM will be identified on T cells which could potentially bind hIgM. Members of the glycan-binding galectin family associate with human T cells^43^,^44^,^45^, and galectin 9 has been shown to bind to the heavy chain of serum IgM^46^. Investigating the expression of hFCMR, or by performing immunoprecipitations using defined glyco-variants of IgM molecules, could help to clarify whether other receptors are indeed involved in IgM internalization by T cells.

Intriguingly, we also showed that IgM potently inhibits T cell proliferation, whereas there was no intrinsic proliferative ability with hIgM alone, supporting the work from previous observations^37^. This finding strongly supports the use of recombinant IgM-based therapies for the treatment of pro-inflammatory conditions, and provides a theoretical underpinning for efforts to harness the therapeutic potential of this under-appreciated class of antibody. Further work now needs to be done to determine the mechanism by which IgM inhibits T cell proliferation.

In conclusion, our findings highlight a critical role for the Cμ4 domain of IgM in binding hFCMR and show that glycans on IgM are not likely involved. This finding is potentially of clinical importance as IgM Fc-fusion proteins are being used to deliver cytotoxic drugs directly to CLL cells via hFCMR^47^. Given the lack of a requirement for IgM glycans in the interaction with hFCMR, these findings suggest that smaller Fc-fusions, encompassing only the Cμ4 domain and lacking sugars, may be more easily manufactured by prokaryotic expression systems for optimal pharmaceutical preparations. As glycans also impede crystallization, our work suggests that it may therefore be possible to remove the glycans from IgM in order to more readily generate co-crystals with hFCMR in structural studies.

## Materials and Methods

### Antibodies and reagents

Anti-human CD4-FITC, anti-human CD19-FITC, goat F(ab’)_2_ anti-human-IgM-RPE, and goat F(ab’)_2_ anti-human-lambda-RPE were from SouthernBiotech. Anti-human CD8-PerCp and anti-human CD56-Alexa-Fluor 700 were purchased from Biolegend. CellTrace Violet Cell Proliferation Kit, LIVE/DEAD Fixable Aqua Dead cell stain kit and anti-human CD3 Brilliant Violet 421 were from Life Technologies, and anti-human FAIM3 (hFCMR, clone 1E4 mouse IgG2bκ) mAb was from Abnova (Taiwan). IgM from human serum, human serum from AB male, and lectin from *Phaseolus vulgaris* (phytohemagglutinin; PHA) were all from Sigma-Aldrich. Biotinylated Sambucus Nigra Lectin (SNA) was from Vector Laboratories, and biotinylated F(ab’)_2_ anti-human IgM was from Jackson ImmunoResearch. The structure, function and purification of domain swapped antibodies have been described previously^16^,^17^,^18^.

### Cells

GFP^+^ hFCMR cDNA-transfected and untransfected mouse BW5147 T cells were generated as previously described^1^. Cells were cultured in RPMI 1640 supplemented with 2 mM L-glutamine, 10% FBS, 50 mM 2-mercaptoethanol, 100 U/ml penicillin and 100 μg/ml streptavidin at 37 °C and 5% CO_2_, with the addition of 1 μg/ml puromyosin to culture medium for the hFCMR cDNA-transfected cells. Buffy coats from blood of healthy individuals were obtained from the National Health Service (NHS) Blood and Transplant Service. Full informed consent and ethical approval for use of human PBMCs was obtained from the Liverpool School of Tropical Medicine ethics committee (reference number 11.92). Consent forms for all volunteer-related human use procedures, such as purifying human PBMCs, are in accordance with the policies established by the institutional review board at LSTM under license 12548 granted by the Human Tissues Authority. Peripheral blood mononuclear cells (PBMCs) were isolated by Ficoll-Paque gradient centrifugation using Lymphoprep (Axis-Shield, Norway) and washed twice in RPMI/10% FBS.

### Glycan analysis

Glycans were removed from hIgM using Peptide-N-Glycosidase F (PNGase F) and α2–3,6,8 neuraminidase according to the manufacturer’s protocol (New England Biolabs). Briefly, 50 μg of hIgM (Sigma-Aldrich) was incubated overnight at 37 °C with the appropriate endoglycosidase and buffers as described previously^39^. Solutions of hIgM and buffer alone were used as controls (non-treated IgM). To determine the success of endoglycosidase treatment, 5 μg of treated or non-treated IgM was separated by 10% SDS-PAGE under reducing and non-reducing conditions, followed by staining with Coomassie Brilliant Blue R250 or immunoblotted with SNA or anti-human IgM (to determine the extent of sialylation and protein loading respectively).

### Molecular dynamic simulations

The atomic model of pentameric hIgM was described previously^27^. The on-line server, Phyre2, was used to identify regions within hFCMR that exhibit high homology to proteins with known structure and then generate homology models of these regions based on these known structures^28^,^29^. In this way, two regions of hFCMR could be modeled with high confidence. One region is homologous (with ~70% confidence) to the transmembrane domain of the ErbB2 receptor tyrosine kinase^30^. The other region is homologous (with 99.6% confidence) to the extracellular, Ig-like domain of IREM-1^48^. Since Ig-like domains of other receptors that bind hIgM are known to directly contact hIgM, we further investigated interactions between this domain and hIgM. In particular, we focused on three loops at one end of this Ig-like domain, the complementarity determining regions (CDR1, CDR2 and CDR3), since these loops directly contact IgM in the other IgM receptors^35^.

We first evaluated two models, Cμ2/Cμ4/hFCMR and Cμ3/Cμ4/hFCMR, using all-atom molecular dynamics simulations to determine which of the two interactions with hFCMR were energetically more favorable. In particular, we manually placed the Ig-like domain of hFCMR within the Cμ2/Cμ4 interface of an IgM monomer (extracted from the pentameric IgM model) or within the Cμ3/Cμ4 interfaces of two neighboring hIgM monomers of the IgM pentamer (Fig. S4). The interaction of hFCMR with the hIgM monomer and dimer were studied, instead of the whole hIgM pentamer, to reduce computational load. The location of this hFCMR domain was chosen such that the CDRs were within close contact with both Cμ domains and that there were favorable contacts between hydrophobic and charged residues on the surfaces of both Cμ and hFCMR domains. For these simulations, we held fixed the positions of residues within the Cμ domains far from the interaction with hFCMR so that the overall structure of the hIgM proteins would be similar to that in the present pentameric hIgM model^27^. For the Cμ2/Cμ4/hFCMR model, the regions held fixed were residues 150 to 165 in Cμ2 domain and residues 322 to 330, 385 to 395, and 415 to 425 in one Cμ4 domain, and residues 338 to 348 and 400 to 410 in the other Cμ4 domain. For the Cμ3/Cμ4/hFCMR model, the regions held fixed were residues 231 to 240, 290 to 300, 323 to 330 and 415 to 425. None of the residues within the hFCMR domain were held fixed. Both models were then solvated in TIP3 water in 0.15 M NaCl and minimized and equilibrated using VMD/NAMD and the CHARMM 27 force field^49^,^50^,^51^. The time step was set at 2 fs, and Langevin dynamics were employed to maintain a constant temperature of 310 K. Equilibration of the systems was evaluated by monitoring the root mean square deviation of the protein backbone. We evaluated the strength of the interaction based on the number of residues within close contact (3 Å) of the binding partner and the extent of buried solvent-accessible surface area, measured using VMD. Finally, we performed equilibration simulations of the interaction between hFCMR and the Cμ2/Cμ4 region with an intact, unconstrained hIgM Fc monomer. We confirmed that the structure of the hIgM Fc monomer at the end of these simulations was compatible with the original model of the pentameric IgM structure.

### IgM binding and internalization

A panel of domain-swap Abs, in which the domains of IgA were replaced with domains of IgM have been described previously^16^,^17^,^18^. These were incubated with hFCMR-transfected cells for 1 hr on ice, washed extensively with ice-cold media and labeled with F(ab’)_2_-anti-human lambda-RPE prior to flow cytometry analysis (BD LSRII).

The ability of hFCMR to internalize hIgM was investigated using a previously described protocol with minor modifications^6^. Briefly, hFCMR-transfected cells or PBMCs were incubated for 1 hr on ice with media alone or media supplemented with 15 μg/ml hIgM or 10% human serum. In some experiments, binding and internalization of endoglycosidase-treated hIgM (15 μg/ml) was also assessed, as was the binding and internalization of heat-aggregated hIgM (62 °C for 30 min)^52^,^53^. Cells were washed twice and then maintained on ice as a control for maximal binding or incubated at 37 °C to allow for receptor internalization. Internalization was halted by the addition of ice-cold media to cells and a brief incubation on ice. Cells were washed and labeled with F(ab’)_2_ anti-human IgM-RPE and/or fluorescent antibodies against human CD3, CD4, CD8, CD19 and CD56 to determine IgM surface levels by flow cytometry.

### Immunofluorescent microscopy

To determine IgM internalization by fluorescent microscopy, 1 × 10^5^ GFP^+^ hFCMR-transfected cells were cytospun onto slides following incubations at 37 °C (as above) and fixed in 4% paraformaldehyde for 10 min at room temperature. Cells were blocked for 15 min in blocking buffer (PBS/5% goat serum), washed with PBS and labeled with F(ab’)_2_ anti-human IgM-RPE in PBS/1% BSA for 1 hr. Following immunostaining cells were washed twice, labeled with DAPI (ThermoScientific) for 5 min at room temperature, and coverslipped. Cell permeabilization was performed in some experiments to determine intracellular levels of IgM following incubations at 37 °C. For this, 0.1% saponin was added to the blocking and staining buffers. Images were acquired using a Zeiss Axioskop microscope using the 40x objective. Images were processed using Abode Photoshop using only level and contrast adjustment (without manipulating the gamma function). The same settings were used for processing and analyzing the images.

### Lymphocyte proliferation assays

PBMCs isolated from healthy donors were labeled with 1 μM CellTrace Violet in accordance with the manufacturer’s instructions. Labeled cells (2 × 10^5^ cells/well) were stimulated with various concentrations of hIgM or BSA pre-incubated with 5 μg/ml PHA for 5d at 37 °C. Cells stimulated with PHA or hIgM/BSA alone were used as positive and negative controls, respectively. After 5d, cells were washed and labeled with anti-human CD4-Alexa Fluor^®^700 and anti-human CD8-FITC to determine cellular proliferation of gated lymphocytes by flow cytometry.

### Statistical analysis

Statistical comparison between various groups was performed by Mann-Whitney comparisons, using the GraphPad Prism 6 software. Differences were determined to be significant when P vales were < 0.05.

### Ethical approval

Buffy coats from blood of healthy individuals were obtained from the National Health Service (NHS) Blood and Transplant Service. Full informed consent and ethical approval for use of human PBMCs was obtained from the Liverpool School of Tropical Medicine ethics committee (reference number 11.92). Consent forms for all volunteer-related human use procedures, such as purifying human PBMCs, are in accordance with the policies established by the institutional review board at LSTM under license 12548 granted by the Human Tissues Authority.

## Additional Information

**How to cite this article:** Lloyd, K. A. *et al*. Glycan-independent binding and internalization of human IgM to FCMR, its cognate cellular receptor. *Sci. Rep.* **7**, 42989; doi: 10.1038/srep42989 (2017).

**Publisher's note:** Springer Nature remains neutral with regard to jurisdictional claims in published maps and institutional affiliations.

## Supplementary Material

Supplementary Information
