# Supplementary material for: Glycan-independent binding and internalization of human IgM to FCMR, its cognate cellular receptor
Source: Sci Rep. 2017 Feb 23;7:42989. doi: 10.1038/srep42989 (PMC5322398; doi:10.1038/srep42989)
Supplement: Supplementary Information [file srep42989-s1.pdf]

Supplementary Information

Glycan-independent binding and internalization of human IgM to FCMR, its cognate cellular receptor

Katy A. Lloyd[1], Jiabin Wang[2], Britta C. Urban[1], Daniel M. Czajkowsky[3], and Richard J. Pleass[1]*.

[1] Liverpool School of Tropical Medicine, Pembroke Place, Liverpool, L3 5QA, UK
[2] Key Laboratory of Systems Biomedicine, Shanghai Jiao Tong University, Shanghai 200240, China
[3] Bio-ID Center, School of Biomedical Engineering, Shanghai Jiao Tong University, Shanghai 200240, China

a.

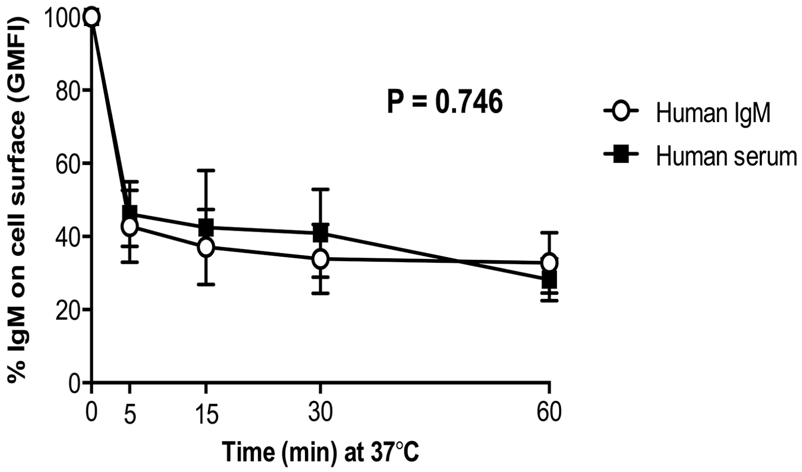

b.

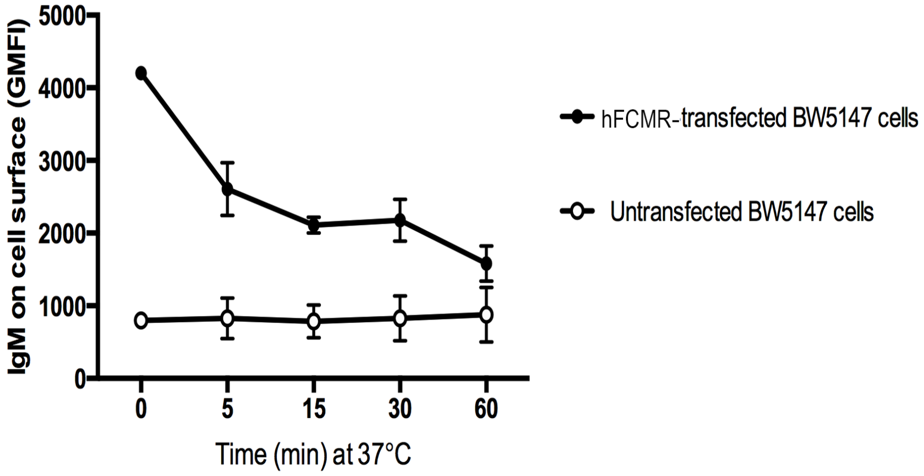

Fig. S1. IgM internalization into hFCMR-transfected cell lines.

(A) Internalization of IgM and human serum (10%) by hFCMR-transfected cells. IgM mean intensity fluorescence (GMFI) shown for indicated time points were normalized to time 0. The mean ± SD from five independent experiments is shown. Mann-Whitney test was used for the comparison of % IgM on cell surface (GMFI) values for hIgM and human serum. Statistical significance was regarded as $P<0.05$. (B) Internalization of hIgM by untransfected and hFCMR-transfected BW5147 cells (n=3).

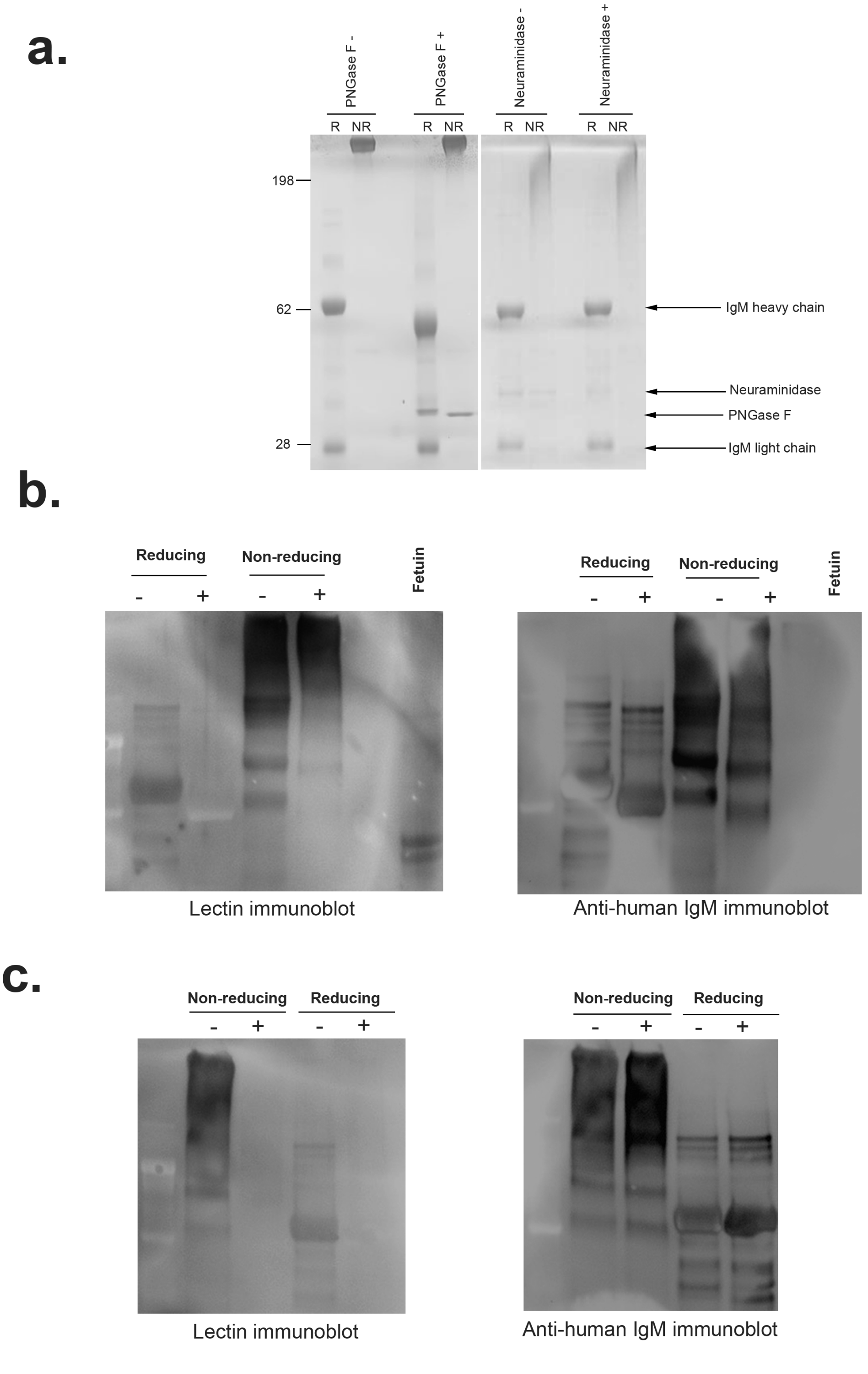

Fig. S2. Glycan analysis. (A) Human IgM (50 μg; Sigma-Aldrich) was digested in the presence (+) or absence (-) of 10 μl (5,000 units) of PNGase F or neuraminidase according to manufacturer's instructions (NEB). After over night incubation at 37˚C, 5 μg of each antibody preparation were separated under reducing (R) or non-reducing (NR) conditions by SDS-PAGE. De-glycosylation was assessed by lectin immunoblotting with SNA and an anti-human IgM antibody for PNGase F treated IgM **(B)** or neuraminidase treated IgM **(C)**.

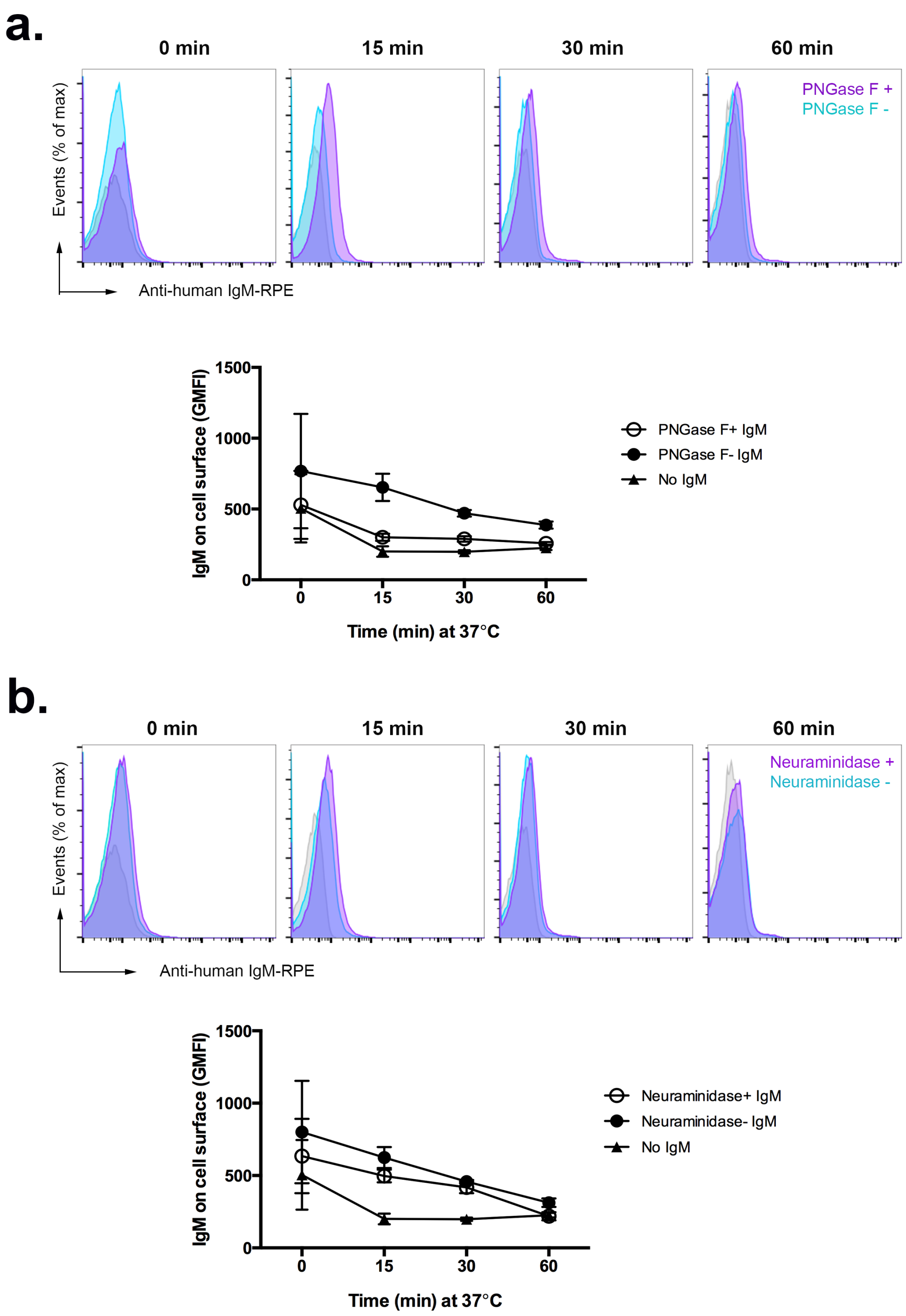

Fig. S3 Internalization of de-glycosylated IgM into untransfected cell lines. Internalization of PNGase F-treated (A) and neuraminidase-treated (B) hIgM by untransfected cells. The mean ± SD of three independent repeats is shown.

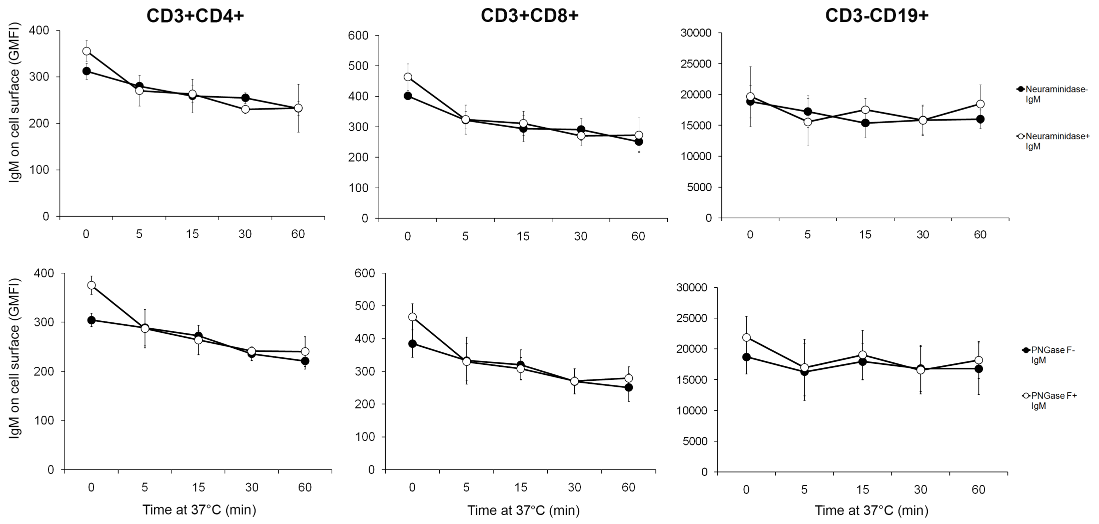

Fig. S4. Effect of de-glycosylation on IgM internalization by lymphocytes.

PBMCs (2 x 10^5) isolated from healthy donors were washed and incubated with media supplemented with endoglycosidase-treated (Neuraminidase+/ PNGase F+) and – non-treated (Neuraminidase -/ PNGase F-) human IgM (15μg/ml) for 1h on ice, prior to internalization assays. Levels of IgM on lymphocyte surfaces (GMFI) were determined by flow cytometry using antibodies specific for CD3, CD4, CD8, CD19, and human IgM. The mean ± SD of three independent repeats is shown.

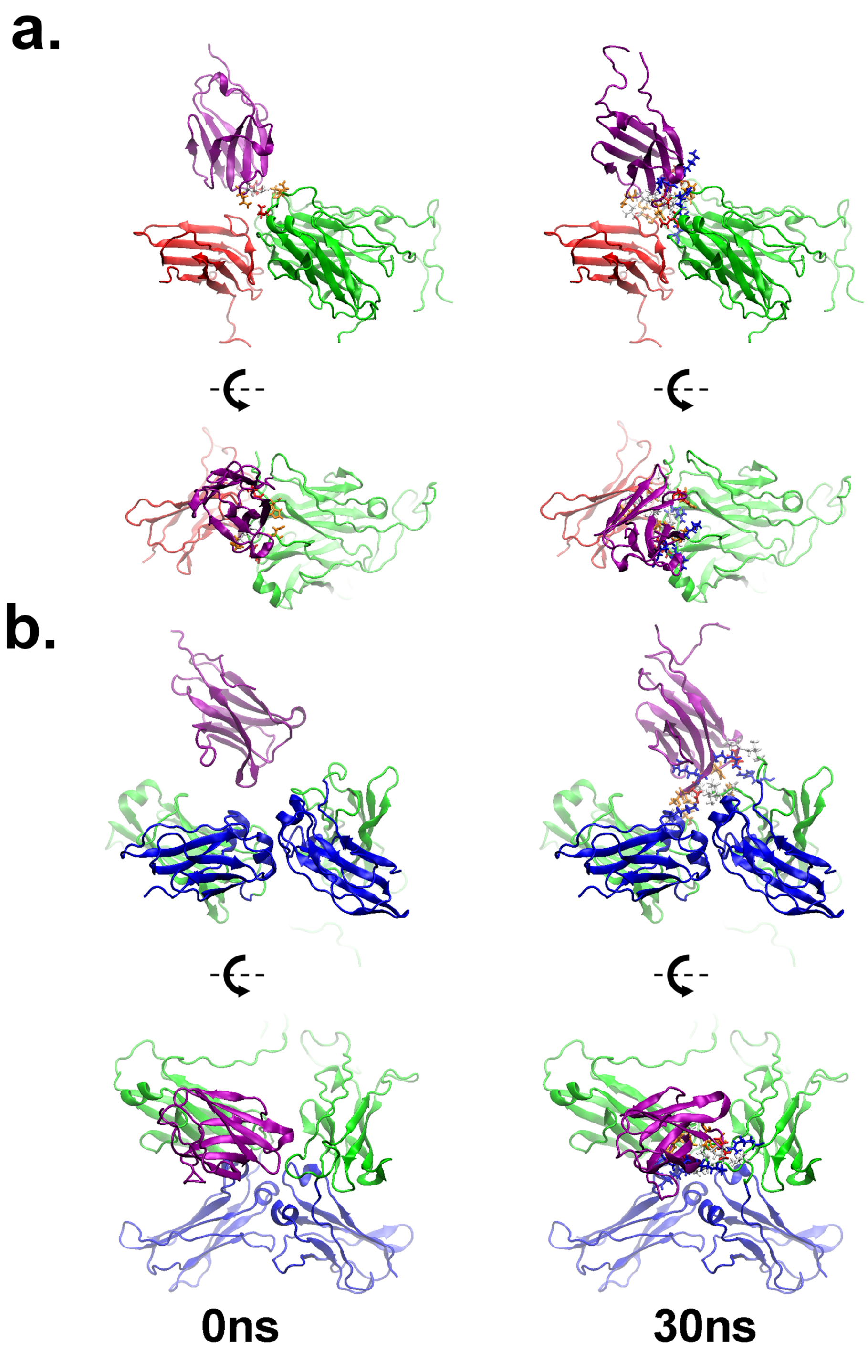

Fig. S5. Evaluation of the Cμ2/Cμ4/hFCMR and Cμ3/Cμ4/hFCMR complexes
(A) The Cμ2/Cμ4/hFCMR model consists of only one Cμ2 and two Cμ4 domains from a single IgM monomer extracted from the pentameric IgM structure. (B) The Cμ3/Cμ4/hFCMR model consists of two pairs of Cμ3/Cμ4 domains from two adjacent IgM monomers extracted from the pentameric IgM structure. Although the contact areas of the initial structures of both the Cμ2/Cμ4/hFCMR and Cμ3/Cμ4/hFCMR complexes were similar, the area of the Cμ2/Cμ4/hFCMR interface was significantly greater than the Cμ3/Cμ4/hFCMR interface at the end of the simulations (30 ns). For these simulations, IgM regions distant from the hFCMR interface were held fixed (see Methods). The proteins are colored as described in Fig. 5.

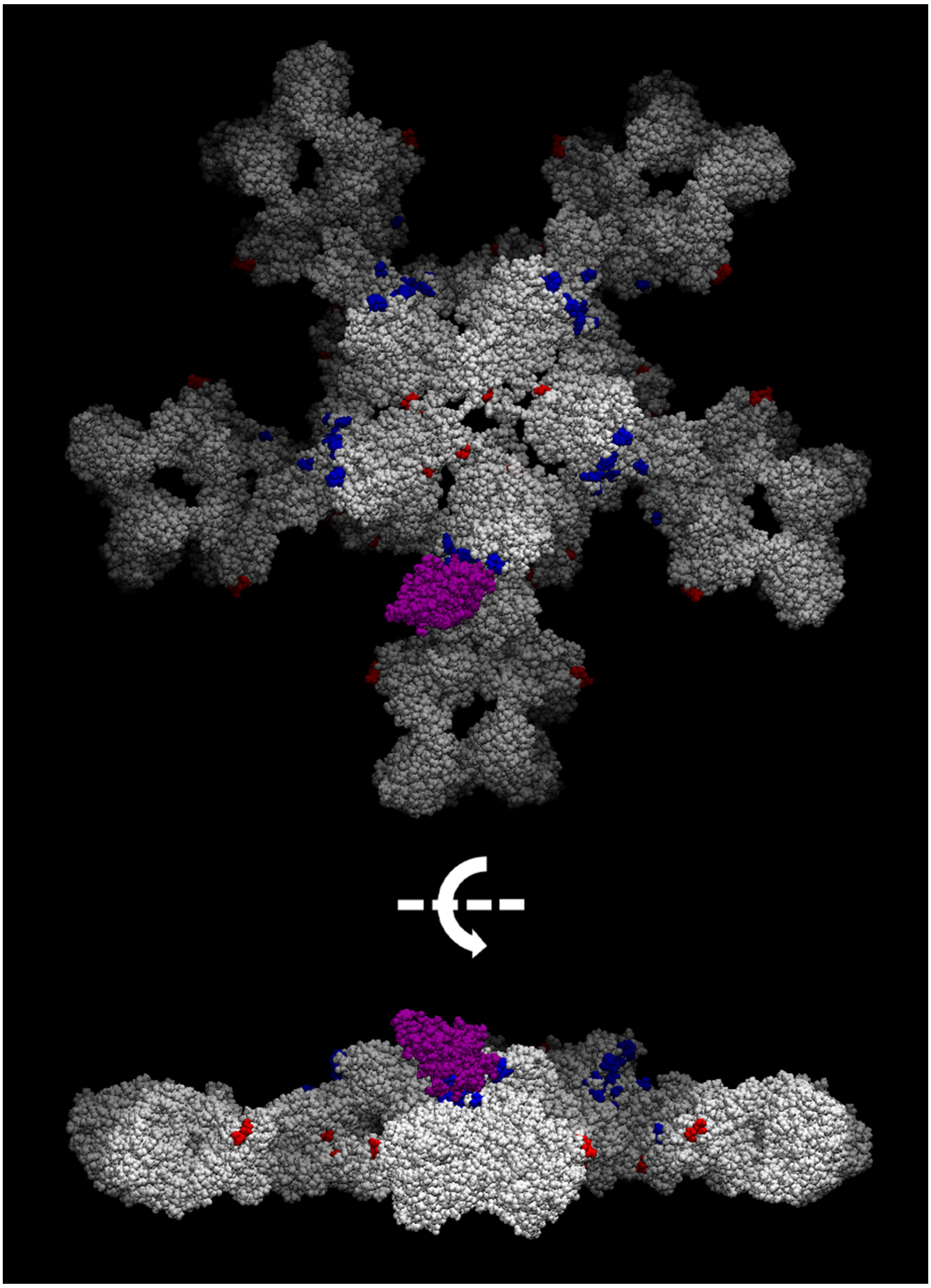

Fig. S6. Model of the hFCMR/pentameric IgM complex.
Shown is the complex of FCMR bound to the pentameric IgM complex based on the model of FCMR bound to the IgM monomer (Fig. 5). All proteins are shown as van der Waals spheres. The IgM pentamer is white, the FCMR is purple, and the residues directly involved in the interaction are blue. The Asn residues in IgM that are glycosylated are red. These residues are found to be far from the proposed location of the FCMR-binding interface, consistent with a lack of an effect of N-glycans on the interaction with FCMR.

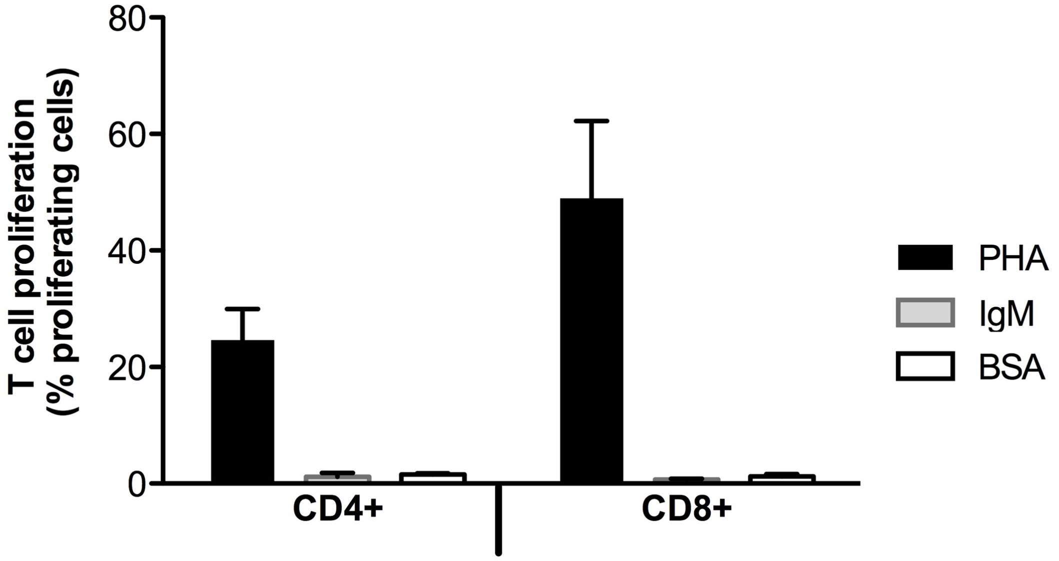

Fig. S7. IgM and BSA do not induce T cell proliferation.

CellTrace™ Violet-labeled PBMCs were incubated with 50 μg/ml IgM or BSA for five days at 37˚C and analysed by flow cytometry. PHA (5 μg/ml) was used as a positive control. Data are represented as percentage of proliferating $CD4^+$ or $CD8^+$ T cells, with error bars depicting the mean ± SD of two independent experiments.
